# Micro RNAs and DNA methylation are regulatory players in human cells with altered X chromosome to autosome balance

**DOI:** 10.1038/srep43235

**Published:** 2017-02-24

**Authors:** Shriram N. Rajpathak, Deepti D. Deobagkar

**Affiliations:** 1 Centre for Advanced Studies, Department of Zoology, Savitribai Phule Pune University, Pune 411007, India; 2 Bioinformatics Center, Savitribai Phule Pune University, Pune 411007, India

## Abstract

The gene balance hypothesis predicts that an imbalance in the dosage sensitive genes affects the cascade of gene networks that may influence the fitness of individuals. The phenotypes associated with chromosomal aneuploidies demonstrate the importance of gene dosage balance. We have employed untransformed human fibroblast cells with different number of X chromosomes to assess the expression of miRNAs and autosomal genes in addition to the DNA methylation status. High throughput NGS analysis using illumina Next seq500 has detected several autosomal as well as X linked miRNAs as differentially expressed in X monosomy and trisomy cells. Two of these miRNAs (hsa-miR-125a-5p and 335-5p) are likely to be involved in regulation of the autosomal gene expression. Additionally, our data demonstrates altered expression and DNA methylation signatures of autosomal genes in X monosomy and trisomy cells. In addition to miRNAs, expression of DNMT1 which is an important epigenetic player involved in many processes including cancer, is seen to be altered. Overall, present study provides a proof for regulatory roles of micro RNAs and DNA methylation in human X aneuploidy cells opening up possible new ways for designing therapeutic strategies.

In mammals, except for genes that escape X inactivation (15%)^1^, the X chromosomal gene dosage gets functionally equalised in male and female cells. Various epigenetic modifications including DNA methylation are important for the X inactivation and numerous other biological processes. DNA methylation is shown to be influenced by sex chromosomal complement^2^,^3^,^4^ and described to be an important factor in gene dosage maintenance. While most chromosomal aneuploidy conditions are lethal, addition or deletion of single (autosome/sex) chromosome leads to Down’s syndrome, Turner’s syndrome and Klinfelter’s syndrome. In such chromosomal aneuploidy, genome wide DNA methylation^5^,^6^,^7^,^8^ as well as gene expression^9^,^10^,^11^ patterns are shown to be get affected. These disorders are associated with characteristic physiological, neuropsychological features including short stature, ovarian dysfunction, osteoporosis, cardiovascular, renal disorders, and predisposition to diabetes mellitus type II, etc. Various reports have shown that in case of human X monosomy, cells have altered gene expression as well as DNA methylation in comparison with normal condtion^6^,^11^,^12^ and are associated with Turner phenotypes. Overall, this implies a complex interrelationship between sex chromosome and autosome with respect to regulation of gene expression as well as epigenetic signatures in mammals.

MicroRNAs are single-stranded RNAs which play a crucial role in the regulation of development, proliferation, differentiation, apoptosis, stress and are associated with several disease conditions^13^. Generally, miRNAs have been implicated in the regulation of post-transcriptional processes; however novel roles for miRNAs in regulation of transcription have recently been suggested^14^,^15^,^16^,^17^. Various recent reports have described the interconnectivity of multiple non-coding RNA-microRNA pathways^18^ and association with multiple diseases including cancer, autism, obesity and Alzheimer disease^19^. In addition, expression of miRNAs has been shown to be regulated by non-coding trans-regulatory RNAs which provides links to multiple common human disorders^20^. Further, promoter methylation and histone acetylation^21^,^22^ are also involved in regulation of miRNA expression while few microRNAs themselves can regulate the epigenetic machinery^23^. Small RNA mediated DNA methylation regulation i.e. RNA-directed DNA methylation (RdDM) has been extensively analyzed in *Arabidopsis thaliana* and shown to be associated with various biological functions^24^. However, the involvement of miRNAs in regulation of DNA methylation in mammalian systems remains a debatable issue. A study on Klinfelter’s (47,XXY) syndrome has reported altered expression of several miRNAs^25^ but no reports are available for human X monosomy and trisomy conditions.

In the present study, by employing high throughput NGS technology, we have determined the miRNAs expression profiles in human untransformed fibroblast cells. These cells possess different number of inactive X chromosomes with 45,X having no inactive while 46,XX and 47,XXX with one and two inactive X chromosomes respectively. Several miRNAs were seen to be differentially expressed and appear to target genes which show altered expression levels under X monosomy^11^. Both miRNAs and DNA methylation are important epigenetic players and it is important to unravel their coordinate expression. We have assessed the alteration in DNA methylation status and gene expression, in X monosomy and trisomy cells in comparison to normal cells (46,XX). Our data shows that altered X chromosomal number modulates autosomal gene expression and DNA methylation. Additionally our data also confirms that the enzymatic machinery for DNA methylation is altered in these cells. Hence the present investigation identifies the regulatory role of micro RNA as well as DNA methylation in human X aneuploidy cells.

## Results

In current study, human untransformed fibroblasts with the 45,X; 46,XX and 47,XXX karyotype were employed. Initial analysis for Xist expression showed that Xist is not expressed in 45,X cells, had moderate expression in 46,XX and highest expression in 47,XXX (P < 0.05, Supplementary Figure S1A) cells. Since Xist expression is associated with X inactivation, this observation correlates and confirms the presence of one and two inactive X chromosomes respectively in 46,XX and 47,XXX cells.

### Small RNA sequencing data generation in human X aneuploidy fibroblast cells

**Figure 1:**
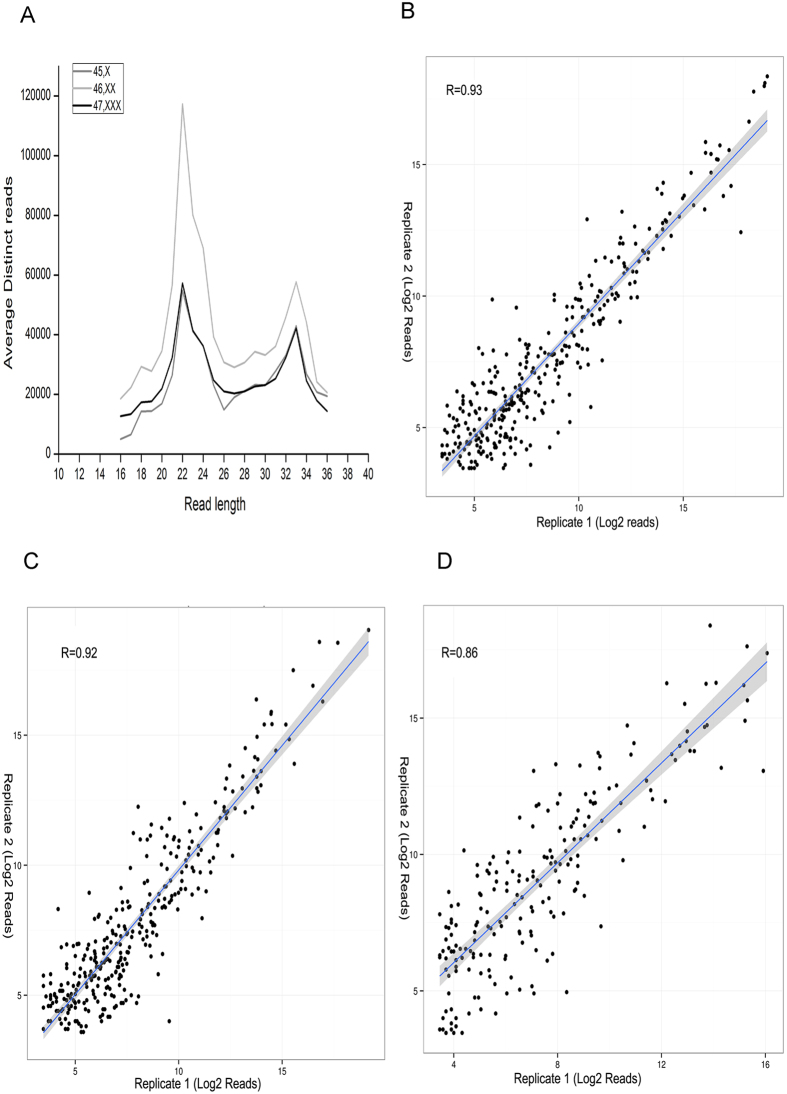
miRNA NGS raw data statistic. (**A**) Read length distribution of mapped reads. **B-D**) Coefficient of correlation for biological duplicate miRNA sequencing in (**B**) 45,X (**C**) 46,XX (**D**) 47,XXX.

Recent reports have implied that miRNAs are important epigenetic regulators. The miRNA profiles in X monosomy and X trisomy conditions are not available. By employing NGS approach, we have determined the sets of miRNAs expressed in the human untransformed fibroblast cells with altered X chromosomal numbers. Small RNA sequencing was carried out in biological duplicates and differential expression of miRNAs was analysed. Small RNA profiling of mapped reads revealed two distinct peaks, one at 21–24 nt position, and another at 31–33 nt position (Fig. 1A). About 13–25 million raw reads were obtained which were further analyzed for the identification of human mature miRNA sequences retrieved from miRbase-21 database^26^. Good correlation was observed between the biological replicates for 45,X (R = 0.92), 46,XX (R = 0.92) and 47,XXX (R = 0.86) data sets (Fig. 1B–D).

### Differential expression of miRNAs in X aneuploidy cells

**Figure 2:**
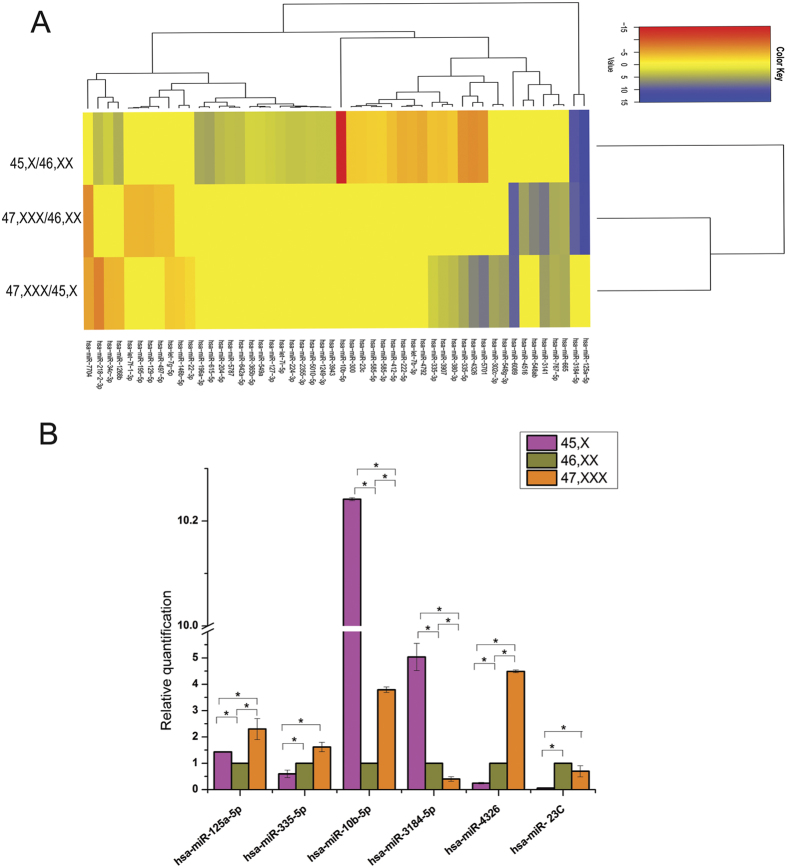
Differential expression of miRNAs. (**A**) Heat map depicting differentially expressed miRNAs (P < 0.05 and Log2 fold change >2). Log2 fold change was used to plot the heat map. (**B**) Validation of MicroRNA NGS data by qRT-PCR expression analysis. All of the miRNAs observed to have significant change in expression in either of the X aneuploidy cell. Experiments were carried out in biological and technical duplicates. Mean ± S.D. values are plotted and significance was calculated by independent sample t-test (P < 0.05).

To identify differentially expressed miRNAs in X aneuploidy (45,X and 47,XXX) in comparison to normal (46,XX) cells, the reads which mapped to miRNAs were converted to reads per million in order to normalize for variations in the number of sequenced reads. Further analysis was carried out by employing the average read numbers of the two duplicates. DGE analysis was carried out considering the replicates using DESeq tool^27^. The small RNAs showing P < 0.05 with log2 fold change >2 were considered as differentially expressed (Fig. 2A).

Several miRNAS (total of 34 miRNAs in 45,X/46,XX; 14 miRNAs in 47,XXX/46,XX and 19 miRNAs in 45,X/47,XXX) were observed to be differentially expressed (Fig. 2A). In X monosomy cells, 11 miRNAs were down regulated while 23 miRNAs were observed to be up regulated in comparison to 46,XX cells. In 47,XXX cells, six miRNAS had lower expression while eight miRNAs were expressed at higher level when compared with normal human 46,XX cells. On comparison of the X trisomy miRNA profiles with those in X monosomy, 12 miRNAs in X monosomy while 7 miRNAs in X trisomy cells were seen to express at lower levels. Most of these differentially expressed miRNAs are encoded by autosomes. Additionally, Few X linked miRNAs like hsa-mir-23c, hsa-miR-222-5p, hsa-miR-224–3p, hsa-miR-767-5p and hsa-miR-6089 were also observed to be differentially expressed. With more stringent criteria (FDR value < 0.05, log2 fold >2 fold) 7 miRNAs in 45,X/46,XX cells, 3 miRNAs in 46,XX/47,XXX cells and in 45,X/47,XXX hsa-miR-5701 showed differential expression (Supplementary Table S1). It can thus be seen that in a normal autosomal background the expression of miRNAs encoded by both autosomes and the X chromosome is differentially regulated in X aneuploidy cells.

### Validation of the differentially expressed miRNAs using quantitative real time PCR (qRT-PCR)

Differential expression of micro RNAs was validated using 6 miRNAs (5 Autosomal and 1 X linked) (Fig. 2B). Except for hsa-miR-10b-5p in X monosomy and miR-3184-5p in X trisomy, all other miRNAs followed the sequencing trend (Supplementary Figure S1B and C).

### Target gene analysis for differentially expressed miRNAs

*In silico* analysis for the differentially expressed (P < 0.05) micro RNAs in 45,X/46,XX cells reveals around 3000 validated gene targets. In case of miRNAs that expressed differentially in 47,XXX/46,XX, around 78 target genes while for differentially expressed miRNAs in X monosomy and trisomy, approximately 2500 validated target genes could be identified (Supplementary Table S2). GO analysis using DAVID database^28^ revealed that, these set of target genes are involved in steroid biosynthesis (P < 0.0006), insulin signaling pathway (P < 0.017) and calcium signalling pathway (P < 0.03). Additionally, these target genes are associated with various biological processes (P < 0.05) such as skeletal system development, blood vessel development, regulation of ossification, sterol metabolic processes, regulation of transcription and ovulation cycle. Some of the related molecular functions (P < 0.05) are transcription factor activity, steroid hormone receptor activity and calcium ion binding (Supplementary Table S2). GO analysis for 11 differentially expressed miRNAs (FDR < 0.05) has identified a set of target genes that are associated with (P < 0.05) steroid biosynthesis, oestrogen signalling, insulin pathway, cancer pathway and are involved in cholesterol biosynthetic, glucose import, oocyte maturation, neuron development, osteoblast differentiation and Insulin signalling processes (Supplementary Table S1).

**Figure 3:**
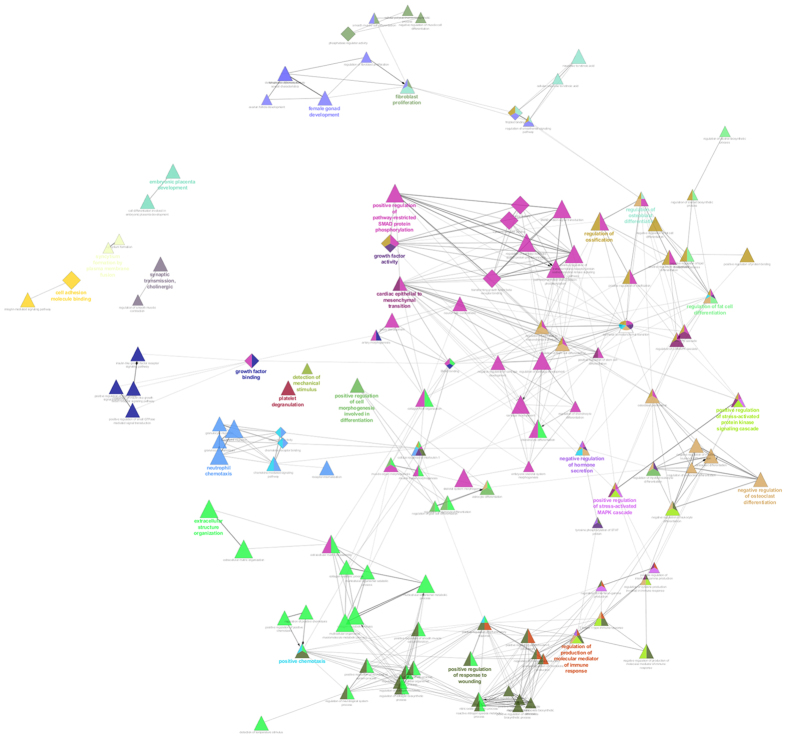
GO analysis for the miRNA target genes. List of differentially expressed gene in X monosomy (P < 0.05) was compared with *in silico* identified targets gene for differentially expressed miRNA in 45,X/46,XX cells. In total 135 genes were found common. The ClueGO app was used to find relevant GO processes for these genes, and a network of connected GO terms was created. Each node represents a GO biological process, and the colours represent the GO group (kappa cut off ≥0.4). Various GO processes including female gonad development and bone metabolism (P < 0.001) were found to be associated with these genes.

Few of these validated targets were observed to be differentially expressed in previous transcriptome data reported by us for 45,X and 46,XX^11^ (Supplementary Table S2). It was observed that nine differentially expressed miRNAs can target 135 differentially expressed genes. Out of these, 69 genes showed high expression and 66 exhibited down regulation in X monosomy in comparison to 46,XX cells. Functional analysis of these genes was carried out by employing Cytoscape ClueGO plugin^29^ (Fig. 3). These set of genes were observed to be involved in several biological process including regulation of ossification, osteoclast differentiation, female gonad development, fat cell development, regulation of hormone secretion and SMAD protein regulation (**P < 0.001, Fisher Exact Test) (Supplementary Figure S2).

**Figure 4:**
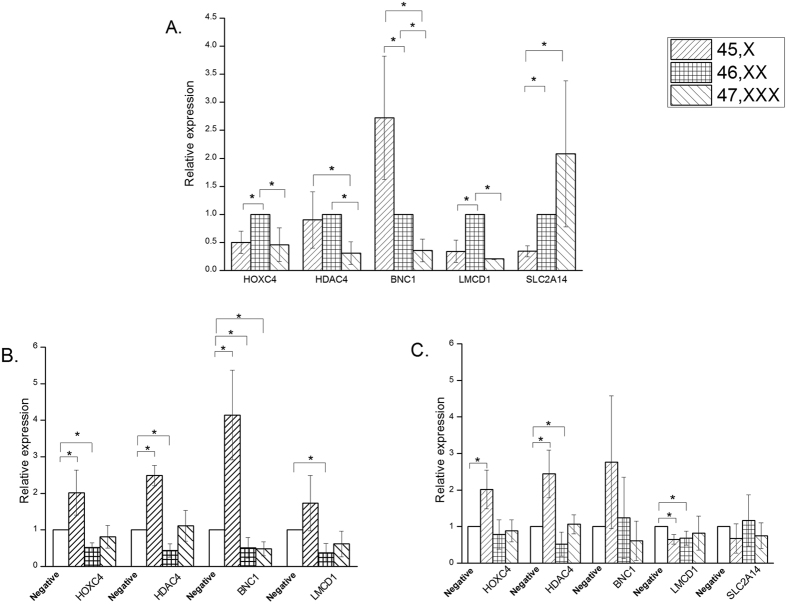
Micro RNA target gene analysis. (**A**) Five identified targets were analysed for differential expression in X aneuploidy cells and showed significant (P < 0.05) change of expression in either X monosomy or trisomy cells in comparison to normal (46,XX) cells. (**B** and **C**) Gene expression analysis of target genes in X aneuploidy cells after transfection of hsa-miR-125a-5p and hsa-miR-335-5p mimics, respectively. Cells with 45,X; 46,XX and 47,XXX karyotype was transfected in three independent experiments with All Stars negative control (mock) and used as control for assessment of gene expression. Target gene expression assays were carried out in biological triplicates and values are plotted as mean ± S.D. Significant change in expression was calculated by independant t-test (P < 0.05).

We have chosen 5 targets amongst which HOXC4, BNC1, LMCD1 and SLC2A14 are common genes from earlier comparative analysis of miRNA targets and RNA sequencing data analysis (Supplementary Table S2). Differential expression of another epigenetic modulator HDAC4 in X aneuploidy cells was also observed in earlier X aneuploidy analysis (unpublished data). Out of these 5 genes HOXC4 is a validated target for miR-125a-5p while BNC1, SLC2A14, LMCD1 are validated targets of miR- 335-5p. Real time analysis showed up-regulation of miR-125a-5p in 45,X and 47,XXX cells and as expected its target gene HOXC4 has low expression (Fig. 4A, Supplementary Table S6). Micro RNA 335-5p was observed to have low expression in X monosomy and corresponding target genes LMCD1, SLC2A14 showed down-regulation while BNC1 showed up-regulation in X monosomy cells (Fig. 4A, Supplementary Table S6). In X trisomy cells miR-335-5p has elevated expression and its target genes BNC1, LMCD1 are down-regulated whereas SLC2A14 showed no change in expression. HDAC4 was seen to have low expression in 47,XXX cells but similar expression in both 45,X and 46,XX cells (Fig. 4A). Gene HOXC4, BNC1 and LMCD1 are transcription factors known to be involved in modulating activity of various other genes. Additionally histone modification enzyme, HDAC4, is a known epigenetic regulator and SLC2A14 is a facilitative glucose transporter.

In order to further validate the specific role of these miRNAs, the transfection experiments were carried out with miRNA mimics and negative control for 45,X; 46,XX and 47,XXX cell karyotype in biological triplicates. Real time PCR analysis showed both miRNA mimics (hsa-miR-125a-5p and 335-5p) have successfully been transfected in all X aneuploidy cells since increased expression (Unpaired t-test P < 0.05) of both miRNAs could be seen as compared to control (Supplementary Figure S3). Transfection of miR-125a-5p mimic affected the expression of validated target gene HOXC4 where in X monosomy it showed increased transcription while in 46,XX cells it was down regulated but there was no effect in 47,XXX cells. Thus gene HOCX4 showed the expected result in normal cells (down expression after transfection) but opposite trend in X monosomy cells (up regulation after transfection) (Supplementary Table S6, Fig. 4B).

Additionally a few of the target genes, namely HDAC4 and BNC1 were observed to be significantly up-regulated in X monosomy whereas LMCD1 showed no effect on expression as compared to mock transfected 45,X cells. In 46,XX cells, expression of HDAC4, BNC1 and LMCD1 genes was observed to be significantly down regulated in comparison to mock transfected 46,XX cells. However, in 47,XXX cells only BNC1 showed low transcription while none of other genes showed change in expression level (Fig. 4B).

Similarly upon transfection of miR-335-5p mimic, amongst its validated target genes, only LMCD1 showed significant down regulation in X monosomy and normal (46,XX) cells but no change in 47,XXX, in comparison to mock transfected cells. However both BNC1 and SLC2A14 had no change in expression in any of the cells with karyotypes 45,X, 46,XX and 47, XXX. Thus in case of miR-335-5p only gene LMCD1 was observed to show the expected results in X monosomy and normal cells. It seems that gene BNC1 and SLC2A14 are not targets of miR-335-5p in the human fibroblast cells used for this study or alternatively a complex interplay of a network of interactions is tuning the gene expression regulation (Supplementary Table S6, Fig. 4C).

Furthermore after transfection of miR-335-5p mimic, gene HOXC4 showed an increased expression in 45,X cells while no alteration in expression was seen in case of 46,XX and 47,XXX cells. HDAC4 showed significantly reduced expression in normal cells and on contrary displayed an increased expression in X monosomy. In 47,XXX cells however, transfection of miR-335-5p mimic did not alter the expression of HDAC4 gene (Fig. 4C).

### Analysis of differential gene expression in X chromosome aneuploidy

**Figure 5:**
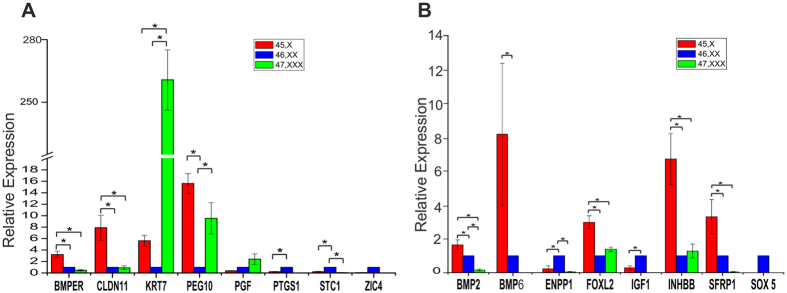
Gene expression analysis. (**A**) TaqMan qRT-PCR expression analysis for the set of genes common in both methylation and RNA sequencing data. (**B**) Expression data for set of other differentially expressed genes. Various gene showed Differential expression with ANOVA post hoc analysis P < 0.05. Gene expression experiments were carried out biological triplicates and values are expressed as mean ± S.E.M.

Our earlier analysis had shown that there is differential gene expression in X monosomy and mis-regulation of DNA methylation appears to be an important epigenetic factor. In the current study, we have analysed gene expression profile in 45,X, 46,XX and 47,XXX cells. We have chosen a set of genes which were differentially methylated^6^ and had differential expression (RNA sequencing data)^11^ in 45,X and 46,XX cells as observed in our earlier studies. Quantitative real time analysis of autosomal genes namely, BMPER, CLDN 11, KRT7, PEG10, PTGS1, STC1 and ZIC4 (Supplementary Table S3) showed significant (ANOVA post hoc analysis P < 0.05) difference in expression in either X monosomy or X trisomy (Fig. 5A) when compared to normal 46,XX fibroblasts. From this set,except for KRT7 and PGF, all other genes including BMPER, STC1, CLDN11 and PEG10 followed the pattern of expression seen in the RNA sequencing data. Genes BMPER (3 fold), CLDN11 (15 fold) and PEG10 (7 fold) showed up-regulation in X monosomy cells as compared to 46,XX and 47,XXX cells. In X trisomy cells, genes KRT7 (260 fold) and PEG 10 (9 fold) show elevated expression, whereas genes PTGS1 and STC1 were down-regulated in X monosomy cells and their expression was below detection limit in 47,XXX cells. The expression of PGF gene was unchanged in either of the X aneuploidy cells. Additionally, gene ZIC4 was observed to be expressed only in normal (46,XX) cells but not in X monosomy (45,X) and trisomy (47,XXX) cells. Comparative analysis of previous data on the methylation^6^ and expression^11^ of 45,X cells revealed that CLDN11 and STC1 exhibited an increased expression while being methylated whereas gene PGF, PTGS1, KRT7, BMPER, PEG10 and ZIC4 showed a negative correlation with methylation.

We have also examined the expression of a few autosomal genes that were not identified as methylated in the methylation microarray, namely BMP2, BMP6, ENPP1, FOXL2, IGF1, INHBB, SFRP1 and SOX5 (Supplementary Table S3). These genes also showed differential expression in 45,X, 46,XX and 47,XXX cells (P < 0.05, Fig. 5B). All 6 genes showed similar expression profile as observed in previous RNA sequencing data while expression of two genes BMP2 and SFRP-1 did not correlate and showed opposite pattern. Interestingly, SOX5 expression was below detectable level in 45,X and 47,XXX cells. Transcription of genes namely, BMP2 (1.6 fold), BMP6 (8 fold), FOXL2 (2.9 fold), INHBB (6 fold) and SFRP1 (3 fold) was higher in X monosomy in comparison to normal fibroblast (46,XX) cells. Except for INHBB and FOXL2 which showed similar expression in 46,XX and 47,XXX all other genes were expressed at very low level in X trisomy. Genes ENPP1 and IGF1 also had significantly (P < 0.05) low expression in X monosomy and negligible expression in X trisomy cells.

Current analysis also includes 7 X linked genes, two (DIAPH2 and FMR1) of which showed differential DNA methylation in genome wide methylome analysis^6^,^30^. The X linked genes localize to the region of X chromosome which is subjected to X chromosome inactivation. As expected none of these X linked genes showed significant change in expression (ANOVA post hoc analysis P < 0.05) in X aneuploidy cells (Supplementary Figure S4). This is consistent with the fact that in 46,XX and 47,XXX cells, the additional X chromosomes are subjected to inactivation. Our analysis thus provides a definitive evidence for altered expression of autosomal genes in either X monosomy or X trisomy as compared to normal 46,XX cells.

### DNA methylation analysis

**Figure 6:**
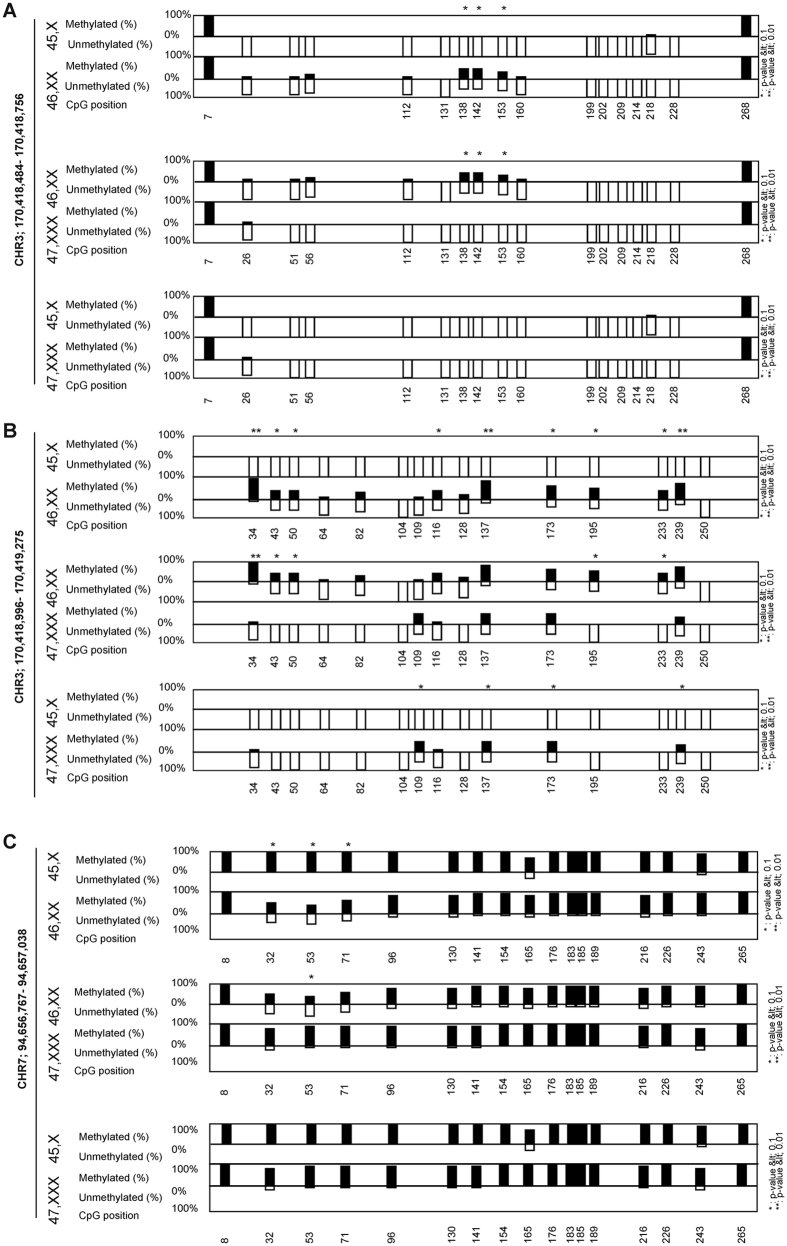
Bisulphite sequencing analysis for promoter region. Data is shown for promoter region of the gene (**A** and **B**) CLDN11 (chr3:170,418,484–170,418,756 and chr3:170,418,996–170,419,275) and (**C**) PEG10 (chr7:94,656,767–94,657,038). Each vertical bar represents single CpG and number below represents CpG position in the selected region. Fisher’s exact test was used for comparing each CpG between two karyotype (*P < 0.1 and **P < 0.01).

DNA cytosine methylation is an important regulatory player. Methylation status of promoter and gene body region for a few candidate genes (four genes namely CLDN11, BMPER, PEG10 and STC1) was analyzed by bisulphite sequencing (hg38 assembly, Supplementary Table S4). For selecting gene body region, probe locations from methylation microarray were retrieved from our earlier study^6^ whereas CpG islands near promoter were analyzed for assessing promoter methylation. Gene STC1 lacks CpG island and hence was not included in promoter methylation analysis. These genes did not show differential DNA methylation in the gene body region (Supplementary Figures S5–S8). Gene BMPER did not show differential methylation (Supplementary Figure S9) whereas both CLDN11 (Fig. 6A and B) and PEG10 (Fig. 6C) showed differential DNA methylation in the CpG sequences near the promoter region. CLDN11 was found to be hypomethylated in 45,X and 47,XXX in comparison to 46,XX cells (Mann-Whitney U-test P < 0.05). Gene PEG10 was observed to have low methylation in 46,XX cells in comparison to 45,X and 47,XXX (Mann-Whitney U-test P < 0.05).

### DNMT1 expression and protein quantification

**Figure 7:**
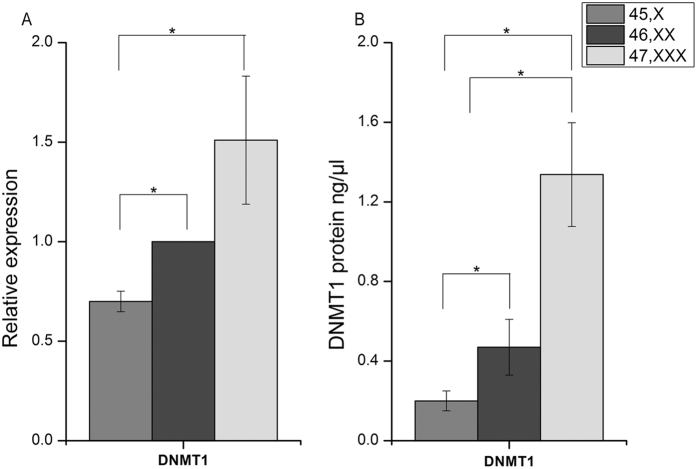
DNMT1 gene expression and protein quantification assay. (**A**) DNMT1 gene expression by TaqMan qRT PCR. (**B**) DNMT1 Protein quantification by ELIZA based commercial assay. Both the results shows that DNMT1 amount is lowest in 45,X compared to 46,XX, 47,XXX (P < 0.05) and was observed to increase in 46,XX followed by 47,XXX cells. Both the assays were carried out in biological triplicates, values are plotted as mean ± S.D. and significance was calculated by independent sample t-test (P < 0.05).

Our earlier observation with 45,X and 46,XX human diploid fibroblast cells suggested that DNA methylation is altered in 45,X thereby providing a molecular genetic explanation for the Turner phenotype. DNMT1 is a maintenance methyltransferase and plays an important role in regulation of DNA methylation signatures. As assessed by TaqMan real time analysis, in comparison to 46,XX cells DNTM1 expression was low in 45,X and highest in 47,XXX (t test P < 0.05) cells (Fig. 7A). Similar to transcription of DNMT1 gene, the protein quantification assay confirms the presences of low amount of DNMT1 in 45,X (0.2 ng/μg), intermediate in 46,XX and highest expression in 47,XXX (1.3 ng/μg) (Fig. 7B). The expression of DNMT1 protein was significantly (Unpaired t test P < 0.05) altered in 45,X, 46,XX and 47,XXX. It is interesting to note that expression of DNMT1 showed a correlation with the number of inactive X chromosomes in these cells i.e. 45,X < 46,XX < 47,XXX.

## Discussion

By employing X aneuploidy cells (45,X and 47,XXX) as a model system, we have investigated the effect of altered chromosomal number on DNA methylation, gene and miRNA expression signatures. Since all X chromosomes in excess of one are inactive due to a process of Lyonisation, these cells possess different numbers of inactive X chromosomes. The gene dosage sensitivity is very well documented in mammals with imprinting effects and lethality associated with most chromosome aneuploidy conditions^31^,^32^. X chromosome has been suggested to have a role in controlling autosomal gene expression and epigenetic signatures^33^. MicroRNAs, have emerged as major epigenetic regulators and are involved in translation, transcription and maintenance of chromosome stability and integrity^34^ including association with various disease conditions. Reports on miRNA expression profiling in autosomal trisomy such as Down’s syndrome^35^, trisomy 13^36^ and trisomy 8^37^ have also identified them as gene expression regulator, biomarkers and possible therapeutic targets in these autosomal abnormalities.

On comparison of miRNA expression profiles from X aneuploidy cells with normal cells (46,XX) it could be seen that X monosomy cells showed higher expression levels for several miRNAs (34 miRNA), whereas cells with X trisomy displayed altered expression of 14 miRNAs. This correlates very well with the observation that X monosomy has severe phenotypes as compared to X trisomy. One interesting observation is that in contrast to mRNA expression, where most of the transcripts were observed to be down-regulated^11^, in case of X monosomy, higher expression of the several micro RNAs (present data) is seen. In addition, 19 miRNAs detected to have differential expression in X monosomy when compared with the X trisomy cells.

Differentially expressed miRNAs in X aneuploidy were observed to target genes associated with glucose metabolism, insulin signalling, steroid metabolism, gonad development, sex determination and bone metabolism pathways. Moreover, these miRNAs are also observed to target sets of genes with transcription regulation, mRNA stabilization and transcription factor activity. These differentially expressed miRNAs have already been shown to be involved in various pathophysiological conditions such as in aneuploidy^38^, obesity^39^, cancer^40^,^41^, Type I diabetes^42^, renal senescence^43^, neural development and differentiation^44^ and also reported to have epigenetic regulatory role^40^,^45^.

X linked miRNAs (P < 0.05), namely hsa-miR-222-5p, 224-3p, 23C, 767-5p and 6089 were also observed to be differentially expressed in X aneuploidy cells. Hsa-miR-222 was shown to have low expression during human adipocyte differentiation^46^ and was suggested as a potential epigenetic therapeutic target for obesity treatment^47^. Also, miR-222-5p has been suggested to have a role in fertility^48^. Another micro RNA miR-224 has been shown to target MBD4 in metastatic colorectal cancer cells^49^. The region transcribing hsa-miR-6089 is located in the PAR region of X chromosome and hence will escape X inactivation. As expected the expression of miR-6089 was observed to be highest in 47,XXX and lowest in X monosomy. Individuals with X monosomy and trisomy display various pathophysiological phenotypes including osteoporosis, diabetes and gonadal failure. In view of the fact that the differentially expressed miRNA (both with FDR < 0.05 and P < 0.05) and their target genes belong to the pathways involved in phenotypes of X aneuploidy, we propose that these miRNAs appear to have regulatory roles. As the current study is restricted to fibroblast cells, it can be speculated that these miRNAs and DNA methylation can serve as molecular biomarkers for X aneuploidy. Their role in embryonic development and lethality could to be analysed by *in vitro* or *in vivo* analysis in cases of X aneuploidy.

Transfection experiments demonstrate that in X aneuploidy miR-125a-5p and miR-335-3p can regulate the expression of a few genes. In X monosomy, both miRNA lead to an increased gene expression (except LMCD1) while in X trisomy and in normal (46,XX) human cells they seem to down regulate the expression of these genes. This analysis not only suggests that miRNA are actively involved in the differential regulation of gene expression but also highlights their distinctive mode of action. Complexity of miRNA regulation is evident as earlier reports have also suggested both inhibition and up-regulation of gene expression by micro RNAs wherein miRNA in specific cells and cell stage can distinctly activate gene translation^50^. Some of the target genes identified in our analysis are transcription regulators (HOXC4, BNC1 and LMCD1) or epigenetic regulators (HDAC4). The altered expression of these miRNAs thus can have grater secondary impact on various downstream genes leading to genome wide changes in gene expression or epigenetic signatures.

Several earlier studies have demonstrated the link between lncRNA and miRNAs regulating gene expression^18^ and association with disease phenotypes^19^. We have previously identified 5 differentially expressed lncRNAs in human X monosomy cells^11^. Further analysis using DIANA LncBase V2 experimental module^51^ led to identification of 4 (miR-10b-5p, miR-125a-5p, miR-4325 and miR-615-5p) differentially expressed miRNAs which can interact with lncRNA Xist. It has been implied that lncRNAs can act as molecular “sponges” of microRNAs, which titrates away the active miRNA affecting the expression of genes^52^. In the cells with varied X/A ratio and inactive X chromosome numbers which have differential expression of Xist, it would be interesting to examine the interaction between these miRNAs and Xist in order to understand miRNA dependant gene regulatory mechanism.

The role of small RNAs in X chromosome dosage compensation has been evaluated in *D. melanogaster*^53^ where X linked siRNAs were seen to promote X-chromosome recognition and dosage compensation. However, in mice^54^ and humans^55^ depletion of Dicer1 did not alter Xist coating on X chromosome. Under these conditions, selective up-regulation of many human X-linked genes^55^ was reported suggesting that DICER depletion may modulate miRNA pathways and thus disrupt the X linked gene expression. On this background, current analysis may help in evaluation of the role of miRNAs, if any, in the maintenance and transmission of information about activity state of the inactive X chromosome during cell division.

Our analysis clearly demonstrates that in X monosomy as well in X trisomy several autosomal genes show altered expression. Genes related to various biological functions which are impaired in X aneuploidy^31^ including bone remodelling (BMP2, BMPER), insulin signalling (IGF2), gonadal development (CLDN11, INHBB) and transcription regulation (FOXL2) were seen to be differentially expressed. This observation thus confirms the association of altered gene expression with X aneuploidy phenotypes.

Bisulphite sequencing confirms that for a set of genes, gene body methylation does not vary while for promoter region of gene CLDN11 and PEG10 significant change in methylation level was observed. CLDN11 is un-methylated in 45,X and has a high level of expression in comparison to 46,XX. Gene PEG10 displays very high expression in 45,X and 47,XXX and is methylated in comparison to 46,XX. Thus for CLDN11 methylation and expression showed an inverse correlation while PEG10 has a direct correlation with promoter methylation.

Interestingly, the expression of DNMT1 protein was lowest in X monosomy cells and increased in 46,XX followed by 47,XXX. Previously Viviana Barra and co-workers^56^ have reported that DNMT1-depletion results in global DNA hypo-methylation and generation of aneuploidy karyotype. In addition, total genomic methylation was shown to increase with inactive X chromosome in the X aneuploidy cells^57^, similar to DNMT1 expression observed in current analysis. The alteration in the expression of epigenetic regulators namely DNMT1 and HDAC4 in X aneuploidy is an interesting observation. This needs to be further validated for regulatory role, if any, in human cells with altered X chromosome number.

Overall the present study reports the influence of altered X chromosomal number on autosomal DNA methylation and in the regulation of mRNA and micro RNA expression. In addition to identifying important molecular players and pathways, this analysis can help in designing novel therapeutic strategies for modulation of phenotypes associated with aneuploidy. In conclusion, current analysis provides a proof of the concept for a regulatory role of DNA methylation and miRNA in X aneuploidy cells, there by opening new roads in X aneuploidy research.

## Material and Methods

### Cell maintenance

Human fibroblast cell lines (white population) with karyotype 45,X (Cat. no. NA 00857) 46,XX (Cat. no. ND 29194) and 47,XXX (NA 04626) were obtained from Coriell Cell Repositories, USA. All fibroblasts were maintained in Dulbecco’s Modified Eagle’s Medium (DMEM, Invitrogen, USA) + 10% fetal bovine serum (FBS, GIBCO), and Penicillin (100 U/ml)- Streptomycin (100 ug/ml) (Invitrogen, USA). Cells were grown at 37 °C with 5% CO_2_.

### Small RNA sequencing

Total RNA was extracted using the Trizol reagent followed by Small RNA enrichment using mirVana PARIS (Ambion), as per manufacturer’s instruction. Sequencing library was prepared as described (TruSeq, Small RNA Kit,Illumina, San Diego, USA). Briefly, 3′ adaptors were ligated to the specific 3′OH group of micro RNAs followed by 5′ adaptor ligation. The ligated products were reverse transcribed with Superscript III Reverse transcriptase. The cDNA was enriched, barcoded by PCR (15 cycles) and cleaned using polyacrylamide gel. The (140–160 bp) library was purified and checked on Agilent 2100 Bioanalyzer (Agilent Technologies, Böblingen, Germany). The sequencing of the library was carried out by using Nextseq500 (Illumina, San Diego, USA). Each sample was sequenced to obtain 75 bases long single reads from the 5′-ends. Sequencing was carried out in biological duplicates. Raw data is available at SRA with accession number ‘SRP097595’

Raw reads were further analyzed to trim the Trueseq adapter sequences and length range filtering (minimum length 16 bp and maximum 36 bp) by using srna-workbenchV3.0_ALPHA^58^. Reads were assessed to generate unique read count profile and these unique reads of length >=16 bp and <=36 bp were further used for identification of miRNAs using *Homo sapiens* mature miRNA sequences retrieved from miRbase-21 database. DESeq Sequencing was carried out in biological duplicates and DGE analysis was carried out considering the replicates using DESeq tool. For further analysis, miRNAs with log2 fold change >2 and P value < 0.05 in expression were employed.

### miRNA target analysis

Using two datasets with experimentally validated targets for miRNAs, namely miR TarBase^59^ and Tarbase V 6.0^60^, possible targets of the differentially expressed miRNAs were identified. Target genes common to both data sets were considered as probable targets. Gene ontology analysis for this dataset was carried out using DAVID database^28^.

### Small RNA sequencing data validation

RNA was isolated from 45,X, 46,XX and 47,XXX cells using miRNeasy kit (Qiagen- 217004), quantified on Nanodrop-Spectrophotometer (Thermo Scientific) and 1 μg of total input RNA was used for cDNA preparation using miScript II RT Kit (Qiagen- 218161) following the manufacturer’s instruction. QRT-PCR was carried out using miScript SYBR Green kit (Qiagen- 218073) and miScript primer assays for detection in the *StepOnePlus* Real-Time PCR System (*Applied Biosystems*). Each micro RNA was analysed in biological and technical duplicates, using SNORD61_11 as an internal control. Statistical analysis was performed with independent sample t-test using GraphPad Software. A total of 6 miRNAs (FDR < 0.05) were selected for qRT-PCR validation (Supplementary Table S5).

### Transfection of micro RNA mimics and target validation

Human cells with following karyotype 45,X;46,XX and 47,XXX were seeded (1 × 10^5^) in 6 well plates 24 hrs before transfection. These cells were transfected with Qiagen micro RNA mimics for hsa-hsa-miR-125a-5p (MSY0000443), hsa-miR-335-5p (MSY0000765) and All Stars negative control siRNA (1027281) using HiPerFect transfection reagent (Qiagen Cat. No.301704). Cells were harvested for RNA isolation after 48 hrs of transfection using miRNeasy kit (Qiagen) and cDNA was prepared using miScript II RT Kit (Qiagen). Another aliquot of the total RNA has used for preparing c-DNA using High Capacity cDNA Reverse Transcription Kit (Cat. No. 00278320, Applied Biosystems) which was used for target quantification by QuantiTect Primer assays (Qiagen) using SYBR Green based qRT-PCR. All the transfection experiments were carried out in biological triplicates. Statistical analysis was performed with independent smaple t-test using GraphPad Software.

### Gene expression analysis

Total RNA was isolated by standard trizol technique and cDNA prepared using SuperScript III, (Invitrogen,USA). Experiments were carried out in biological triplicates using TaqMan assays with 18S rRNA as an internal control. Statistical analysis was performed using one way ANOVA with post hoc analysis by Mega Stat software. Genes with values of P < 0.05 were considered to be significantly altered in expression.

### Bisulphite conversion of genomic DNA

Purified genomic DNA (isolated from cells at passage 11–12) was subjected to bisulphite conversion using Methyl Easy Xceed kit from Human Genetic Signatures (MEOO2), according to manufactures instructions. Bisulphite primers were designed using ZYMO Bisulphite Primer Seeker 12S. Amplified PCR products were cloned in to the *pGEM-T Easy* Vector Systems (Promega) and 8 to 12 positive clones were analysed by sequencing for each PCR product. Differential DNA methylation was analysed by online software Quantification tool for Methylation Analysis^61^.

### DNMT1 protein quantification

Total protein extract from cells was prepared using RIPA buffer (Sigma) and used for *in vitro* DNA methyltransferase-1 protein quantification by EpiQuik Dnmt1 Assay Kit (Epigentek, P-3011-3). Initially the standard curve was generated with purified DNMT1 protein (provided by manufacturer) and the protein amount was quantified from cells extracts (Supplementary Figure S10). Experiments were done in biological triplicates and statistical analysis was performed with independent samples t-test using Graph Pad Software.

## Additional Information

**How to cite this article**: Rajpathak, S. N. and Deobagkar, D. D. Micro RNAs and DNA methylation are regulatory players in human cells with altered X chromosome to autosome balance. *Sci. Rep.* **7**, 43235; doi: 10.1038/srep43235 (2017).

**Publisher's note:** Springer Nature remains neutral with regard to jurisdictional claims in published maps and institutional affiliations.

## Supplementary Material

Supplementary Table S1

Supplementary Table S2

Supplementary Table S3

Supplementary Table S4

Supplementary Table S5

Supplementary Table S6

Supplementary Datsaset 1
