# Supplementary material for: Micro RNAs and DNA methylation are regulatory players in human cells with altered X chromosome to autosome balance
Source: Sci Rep. 2017 Feb 24;7:43235. doi: 10.1038/srep43235 (PMC5324395; doi:10.1038/srep43235)

Micro RNAs and DNA methylation are regulatory players in human cells with altered X chromosome to autosome balance

Shriram N. Rajpathak[1], Deepti D. Deobagkar [1 2 *]

Supplementary Tables:-

Table S1: List of differentially expressed micro RNAs both with FDR<0.05 and P<0.05
Table S2: Micro RNA target gene list
Table S3: Details of the Taqman probe and Syber Green primers used for gene expression analysis
Table S4: Details of the gene regions and primers used for bisulphite sequencing
Table S5: Qiagen assay Ids used for miRNA and target expression analysis
Table S6: Table describing miRNA transfection results for validated target genes

Supplementary Figures:-

Figure S1:- A) Xist expression in 45,X , 46,XX and 47,XXX cells. Xist expression was below detectable level in 45,X and significantly high in 47,XXX (Paired t test P<0.05). Biological triplicate data plotted as Mean ± S.D. B) and C) shows miRNA gene expression and NGS data comparison for 45,X and 47,XXX cells relative to 46,XX cells , respectively.

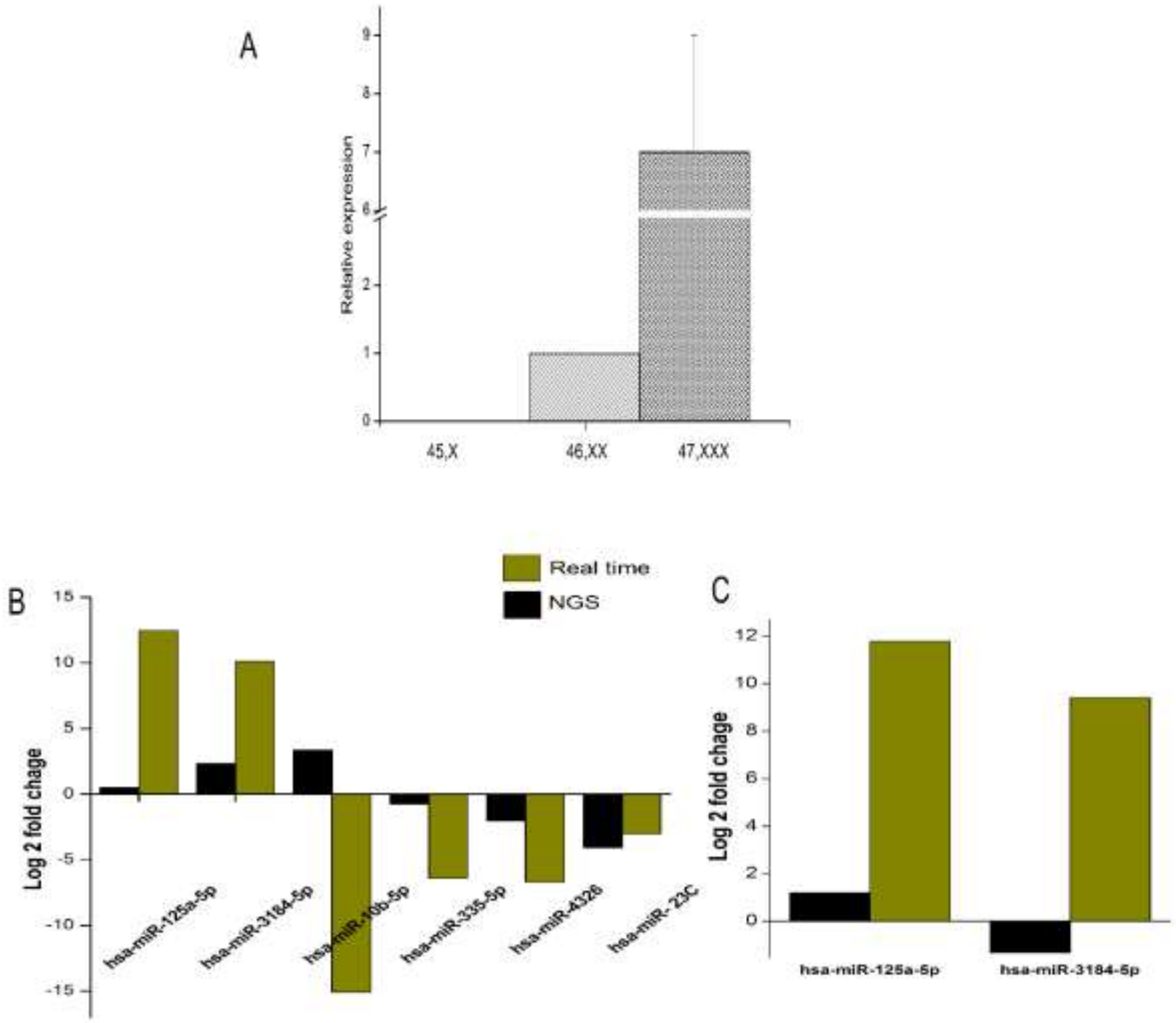

Figure S2:- Pie Chart depicting GO associated with miRNAs target genes from previous RNA sequencing data (** P<0.001)

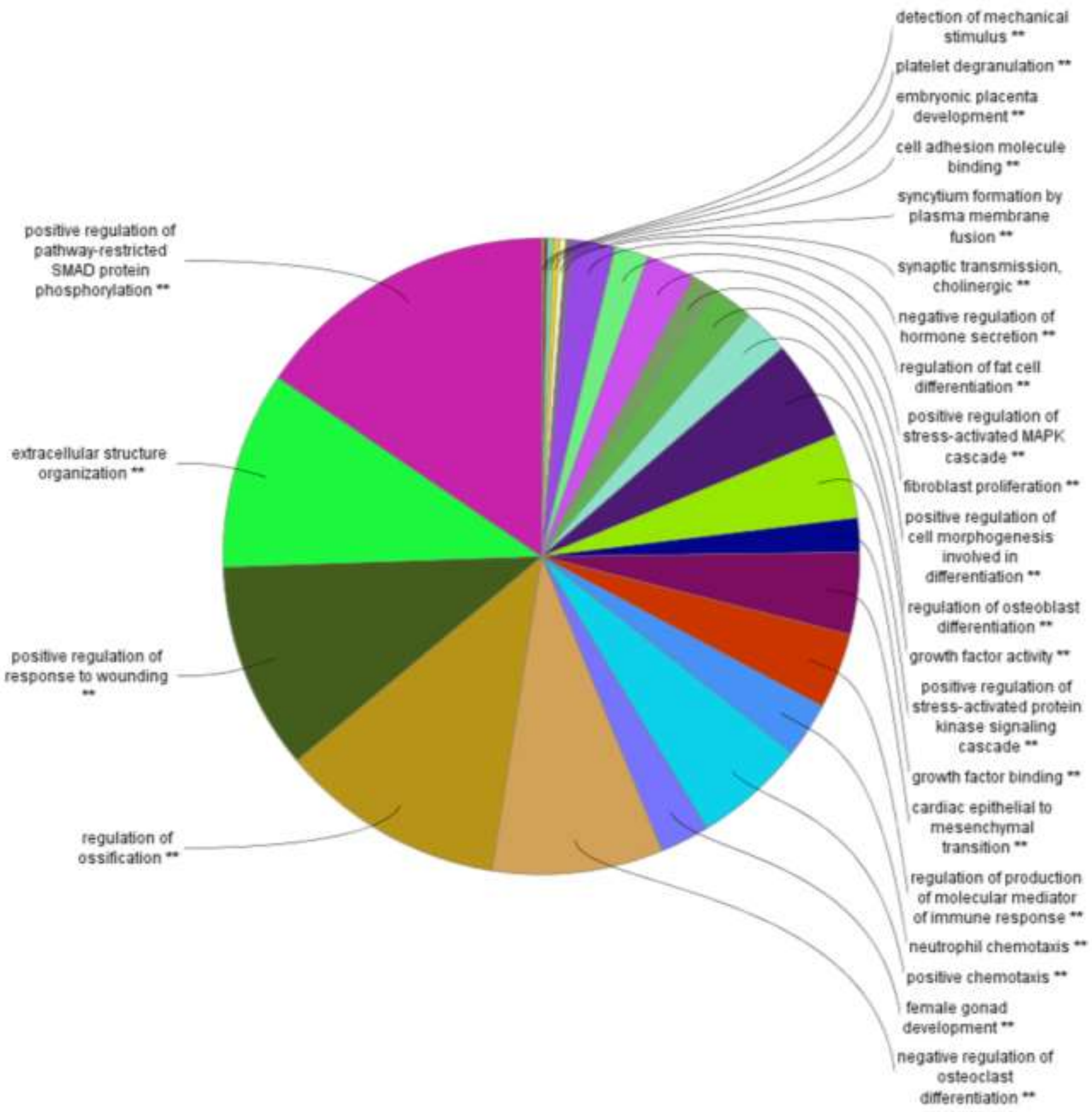

Figure S3:- Relative expression of miRNAs (has-miR-125a-5p and has-miR-335-5p) after mimic transfection in 45,X , 46,XX and 47,XXX cells relative to negative siRNA transfected cells. Experiments were carried out in biological triplicates and average relative expression is plotted. Independent t-test showed significant (P<0.05) up regulation of miRNA in each cell compared to controlled cells depicting successful mimic transfection.

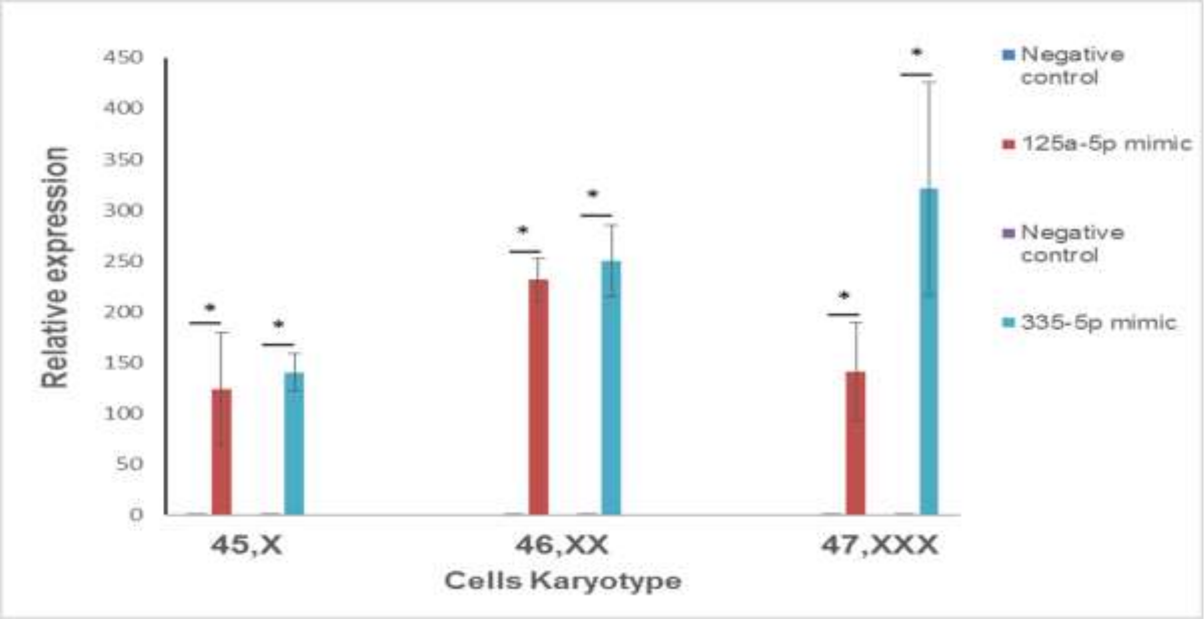

Figure S4:- Expression data for X linked genes

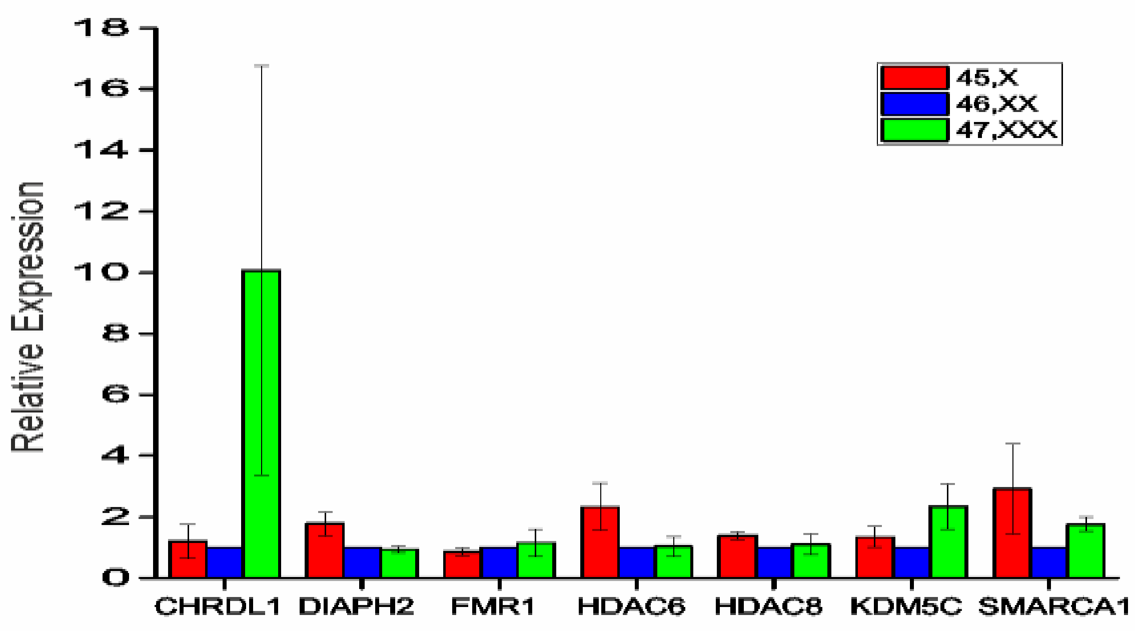

Figure S5:- Bisulphite sequencing data for CLDN11 gene body region

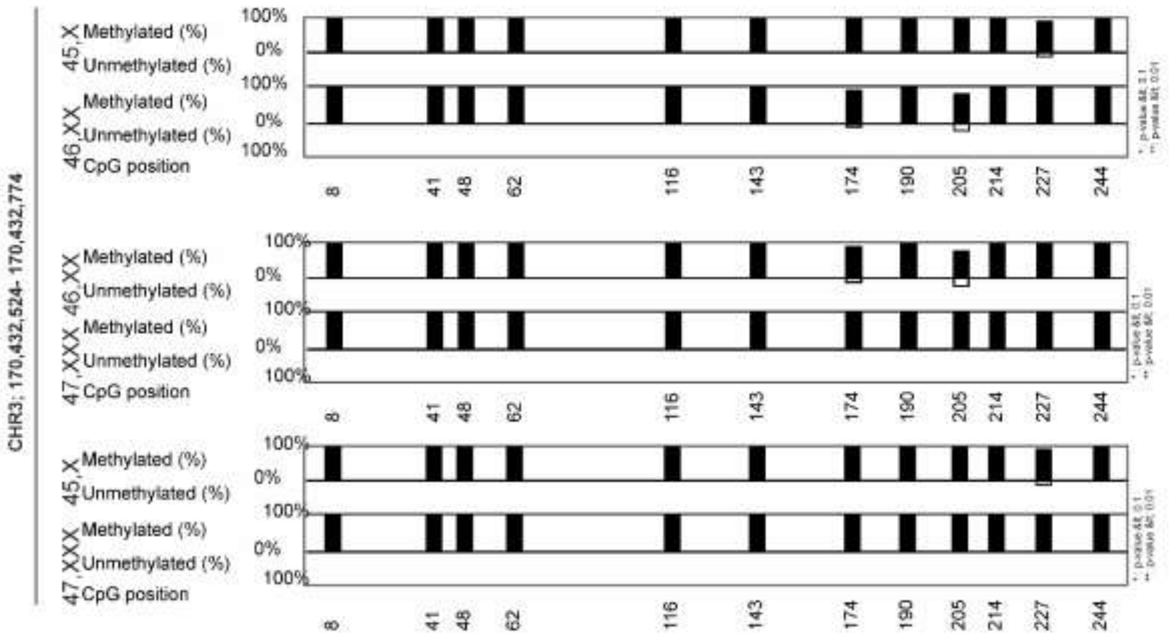

Figure S6:- Bisulphite sequencing data for BMPER gene body region

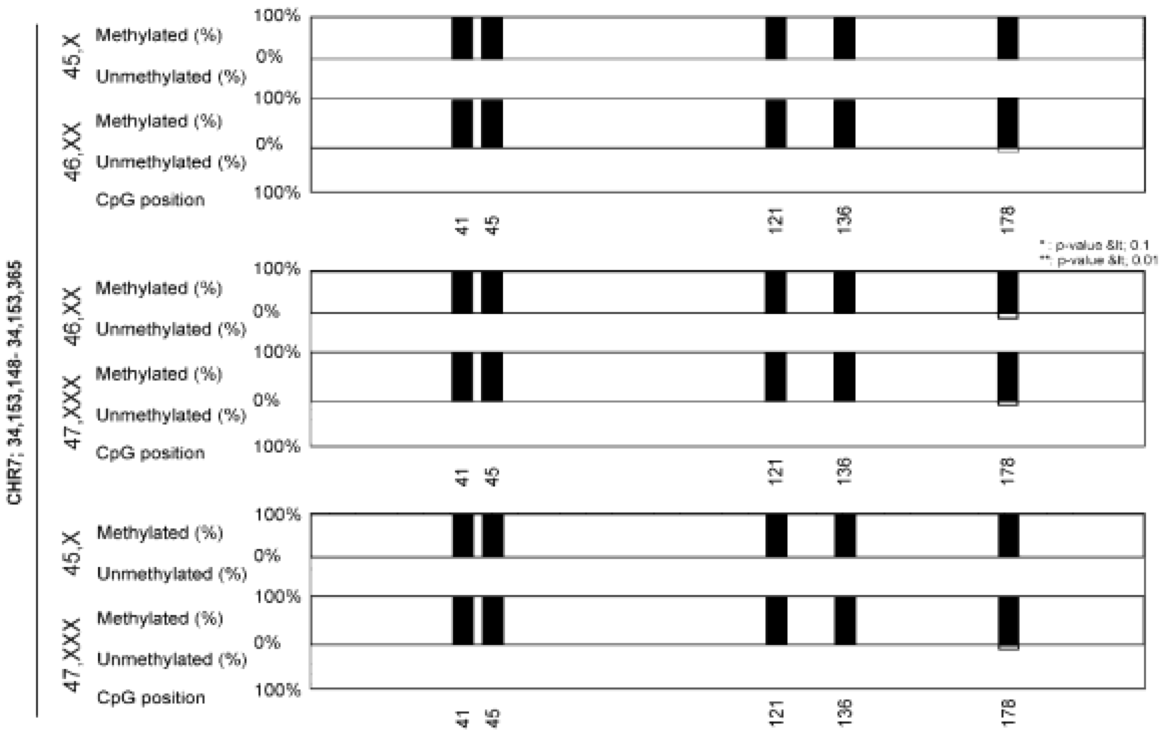

Figure S7:- Bisulphite sequencing data for STC1 gene body region

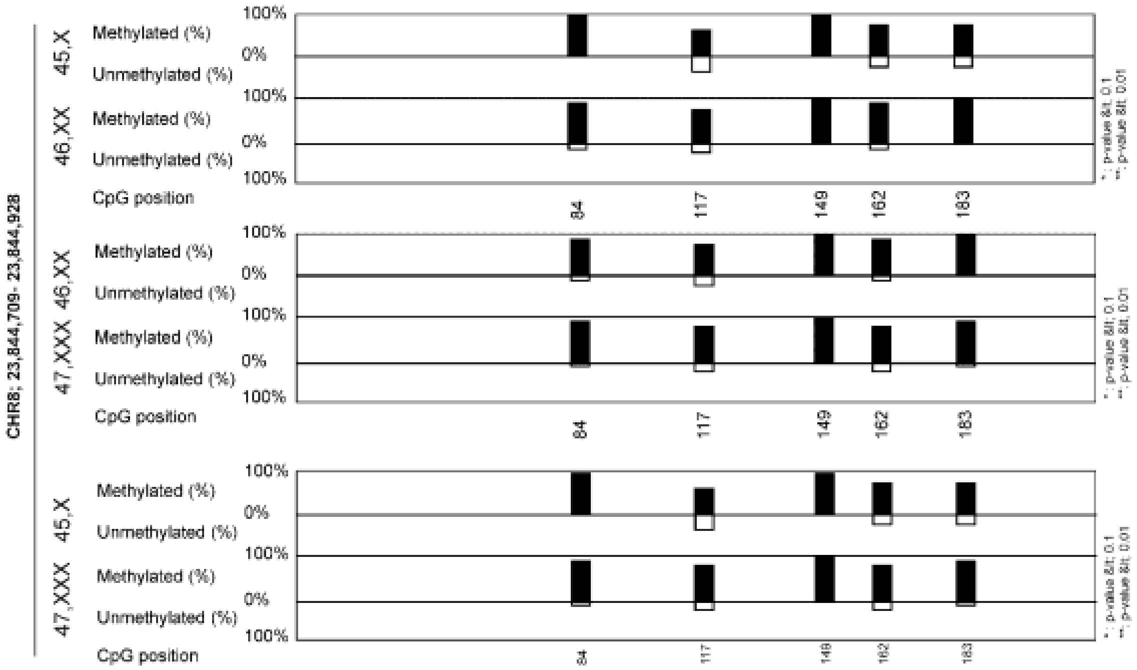

Figure S8:- Bisulphite sequencing data for PEG10 gene body region

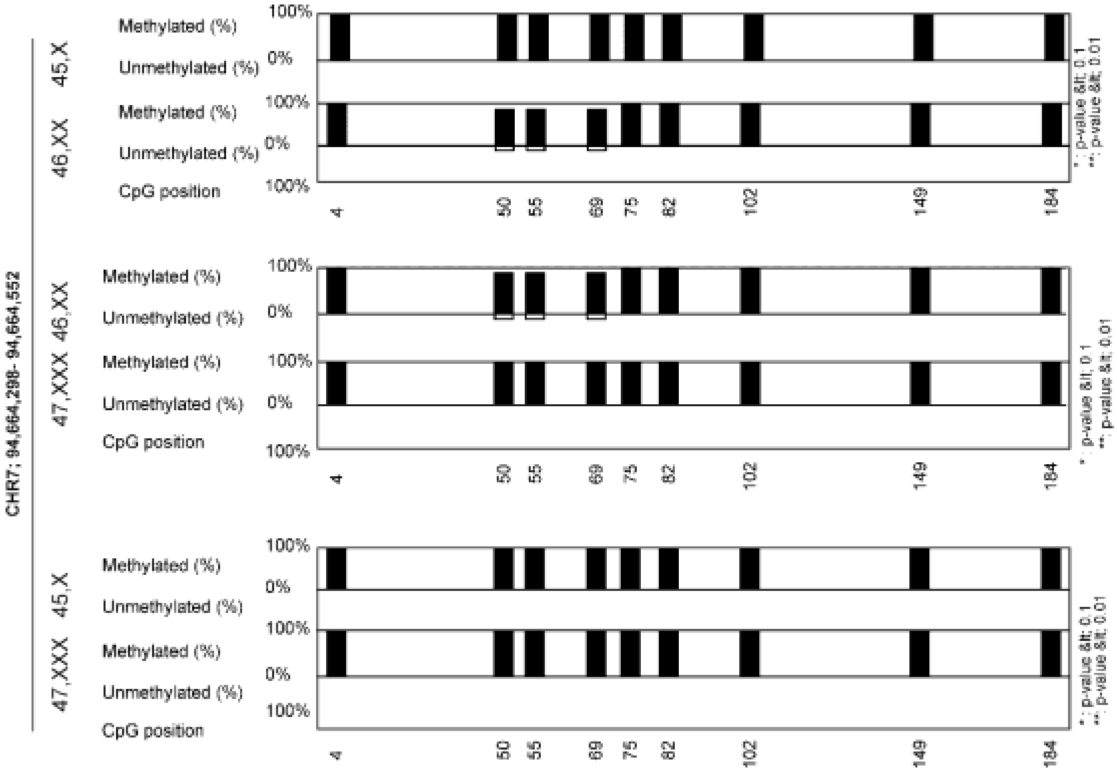

Figure S9:- Bisulphite sequencing data for Promoter region of gene BMPER

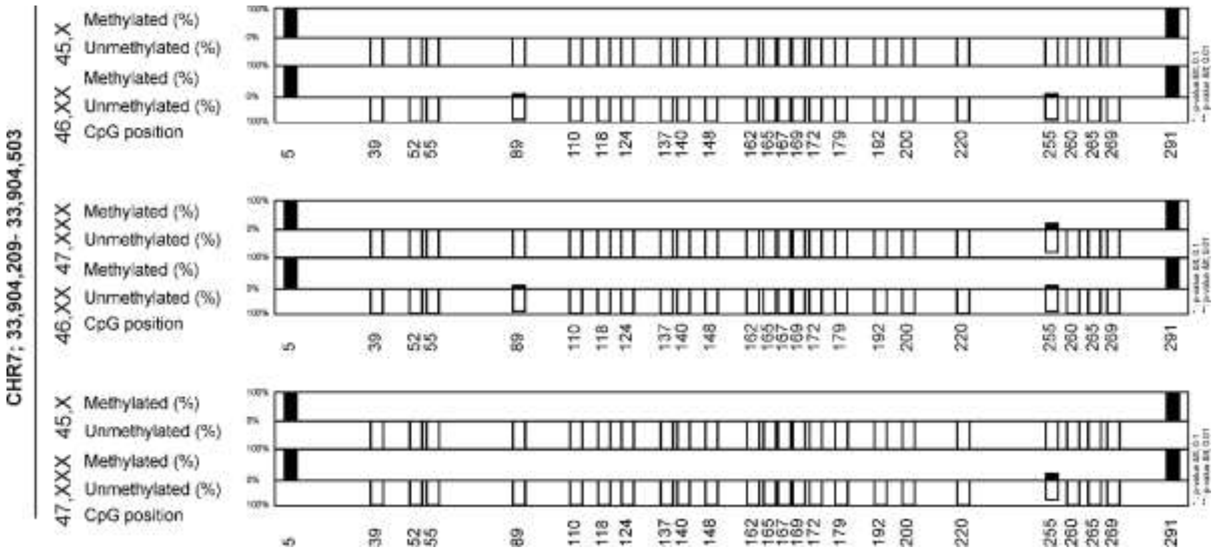

Figure S10:- Illustrate standard curve generated with DNMT1 standard

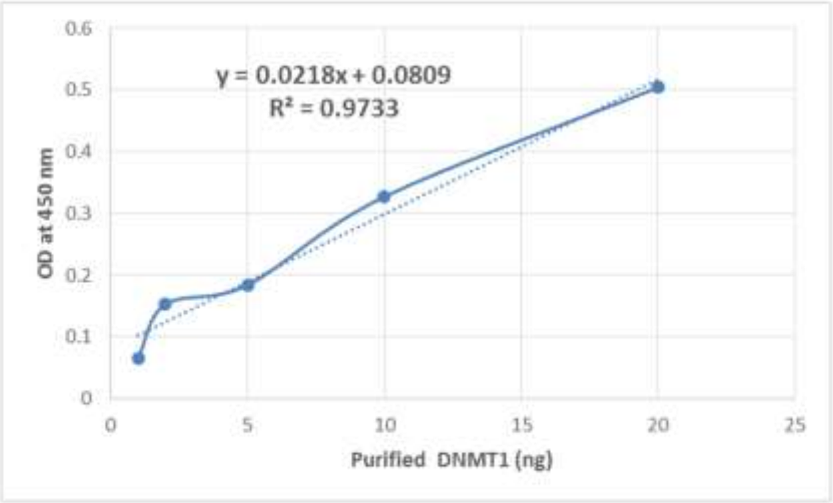

Supplement: Supplementary Datsaset 1 [file srep43235-s7.pdf]
